# A deep learning aided bone marrow segmentation of quantitative fat MRI for myelofibrosis patients

**DOI:** 10.3389/fonc.2025.1498832

**Published:** 2025-05-23

**Authors:** Humera Tariq, Lubomir Hadjiiski, Dariya Malyarenko, Moshe Talpaz, Kristen Pettit, Gary D. Luker, Brian D. Ross, Thomas L. Chenevert

**Affiliations:** ^1^ Department of Radiology, University of Michigan, Ann Arbor, MI, United States; ^2^ Department of Internal Medicine-Hematology/Oncology, University of Michigan, Ann Arbor, MI, United States

**Keywords:** myelofibrosis, proton density fat fraction (PDFF), pelvic MRI, in-phase (IP), proximal femur, posterior ilium, segmentation, U-Net

## Abstract

**Purpose:**

To automate bone marrow segmentation within pelvic bones in quantitative fat MRI of myelofibrosis (MF) patients using deep-learning (DL) U-Net models.

**Methods:**

Automated segmentation of bone marrow (BM) was evaluated for four U-Net models: 2D U-Net, 2D attention U-Net (2D A-U-Net), 3D U-Net and 3D attention U-Net (3D A-U-Net). An experienced annotator performed the delineation on in-phase (IP) pelvic MRI slices to mark the boundaries of BM regions within two pelvic bones: proximal femur and posterior ilium. The dataset comprising volumetric images of 58 MF patients was split into 32 training, 6 validation and 20 test sub-sets. Model performance was assessed using conventional metrics: average Jaccard Index (AJI), average Volume Error (AVE), average Hausdorff Distance (AHD), and average Volume Intersection Ratio (VIR). Iterative model optimization was performed based on maximizing validation sub-set AJI. Wilcoxon’s rank sum test with Bonferroni corrected significance threshold of p<0.003 was used to compare DL segmentation models for test sub-set.

**Results:**

2D segmentation models performed best for iliac BM with achieved scores of 95-96% for the VIR and 87-88% for AJI agreement with expert annotations on the test set. Similar performance was observed for femoral BM segmentation with slightly better VIR but worse AJI agreement for U-Net (94% and 86%) versus A-U-Net (92% and 87%). 2D models also exhibited lower AVE variability (8-9%) and ilium AHD (16 mm). The 3D segmentation models have shown marginally higher errors (AHD of 19-20 mm for ilium and 10-12% AVE-SD for both bones) and generally lower agreement scores (VIR of 91-93% for ilium and 89-91% for femur with 85-86% AJI).

Pairwise comparison across four U-Nets for three metrics (AHD, AJI, AVE) showed that AJI and AHD performance was not significantly different for 3D U-Net versus 3D A-U-Net and for 2D U-Net versus 2D A-U-Net. Except for AVE, for majority of performance metric comparisons 2D versus 3D model differences were significant in both bones (p<0.001).

**Conclusion:**

All four tested U-Net models effectively automated BM segmentation in pelvic MRI of MF patients. The 2D A-U-Net was found best overall for BM segmentation in both femur and ilium.

## 1 Introduction

Myelofibrosis (MF) is a chronic malignancy characterized by clonal proliferation of hematopoietic stem cells. Hallmark features of MF include bone marrow (BM) fibrosis with reticulin or collagen deposits, heterogeneous expression of inflammatory cytokines, anemia, and enlargement of the liver and spleen (hepatosplenomegaly) (1, 2). BM aspirates from ilium with needle biopsies are currently used for clinical MF patient management to assess systemic inflammation, progressive fibrosis and changes in composition. However, these procedures are painful, select limited tissue volume, subject to histopathological sampling errors, and provide inadequate assessment of BM heterogeneity (3, 4). BM heterogeneity measurement in MF patients constantly pose a challenge in hematopathology (3). A typical MF needle biopsy samples small fraction of the area visualized by a pelvic MRI examination which covers >200 cm^2^ of bones encompassing iliac wings, femoral heads, trochanter, and proximal femoral shafts. The non-invasive MRI holds potential for comprehensive MF disease monitoring (3, 5, 6) at multiple anatomic bone marrow sites increasing patient comfort for repeated examinations, anatomic coverage and diagnostic accuracy.

Unlike limited biopsy access, MRI affords large volume coverage for more extensive BM survey that is critical for accurate evaluation of disease heterogeneity at MF primary site (3) and corresponding treatment efficacy during longitudinal monitoring (4–6). Non-invasive assessments of spatially heterogeneous BM pathologies would benefit from the development of robust BM MRI protocols and quantitative imaging biomarkers (QIBs) (7). Quantitative fat MRI is being investigated to assess progressive fibrosis, or reduction in blood cell production due to replacement of normal fat content by cancer cells in MF patients (5). Pre-clinical MF studies (8, 9) also investigate imaging biomarkers to establish quantitative thresholds for comprehensive monitoring of BM disease progression, heterogeneity and therapy response (6, 10). Several promising QIBs are currently investigated for MF bone marrow assessment (5, 8, 9). Proton density fat fraction (PDFF) measures fat content, T2* assesses fibrosis and iron content, apparent diffusion coefficient (ADC) reflects cellular density and microstructural changes, magnetization transfer ratio (MTR) measures the amount of free water within tissue, providing information about tissue composition and pathology, and magnetic resonance elastography (MRE) evaluates soft tissue stiffness, indicating the degree of fibrosis.

Manual delineations of BM within MF patients’ pelvic bones is a major MRI QIB analysis bottleneck both for clinical and pre-clinical studies as they are time-consuming and irreproducible due to varying acquisition parameters, contrasts and intra- and inter-observer biases (11–13). Manual bone annotations are pivotal for detailed regional assessment of QIB histograms, e.g., in the posterior ilium versus proximal femur versus lumber vertebrae. However, they substantially slow down the analysis and are known to introduce inaccuracies due to operator training/bias (11, 12) which consequently have limited clinical acceptance of MF QIBs to date. The annotated data-sets available for training automated BM segmentation models are usually small and protocol-specific (11, 13) presenting a challenge for generalization (14).

The Standard U-Net deep learning (DL) models use coarse-to-fine training strategy, facilitated by skip connections that enable accurate segmentation and reproducible boundary delineation (15, 16) and therefore have shown promise for automating segmentation of quantitative imaging applications with small annotated training sets (14). The latest studies for assessment of spatial distribution of PDFF in vertebral BM also adopted U-Net models (11–13). An Attention U-Net (A-U-Net) enhances the standard U-Net by integrating attention gate mechanisms (17), which focus on relevant regions improving segmentation accuracy, especially for small or complex structures. Our group has recently successfully applied 2D Attention-U-Net (A-U-Net) for tibia segmentation of a preclinical model of myelofibrosis (12). The present study sought to evaluate the viability of 2D and 3D U-Net segmentation models to accurately localize femoral and ilium BM regions on in-phase (IP) image volumes of MF patients.

## 2 Materials and methods

### 2.1 MRI data acquisition and preparation

**Figure 1:**
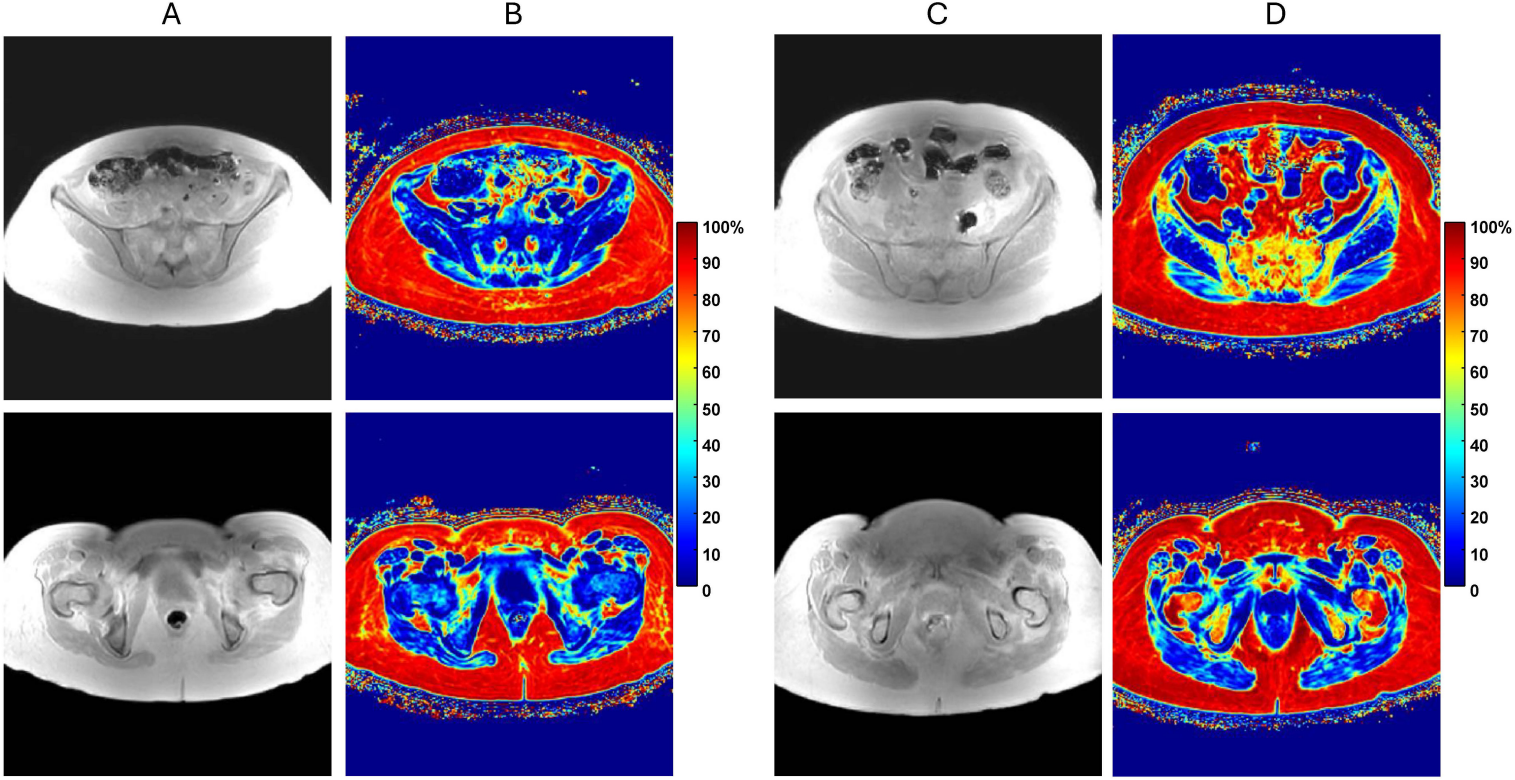
**(A)** In-phase (IP) pelvic MRI of myelofibrosis (MF) patient with low bone marrow fat content for axial slice through ilium (top) and femur (bottom). **(B)** Corresponding quantitative proton-density fat-fraction (PDFF) maps shown on 0 to 100% color scale. **(C)** Similar IP MRI of MF patient with relatively high fat content with **(D)** associated PDFF maps. Note, only IP images were used for segmentation by expert annotator and DL models.

A single center MRI study for IRB-consented MF subjects was performed using standard imaging protocol. All MF patient MR images were acquired using clinical mDIXON-QUANT protocol with images reconstructed on a 3T scanner including quantitative PDFF maps (**Figure 1**). Patient demographics and image counts for each bone site are available in **Supplementary Table S1**. Typical pelvic MRI scanned volume comprised 57 axial slices (3 mm thick) within 400x400 mm^2^ FOV with image size of 288x288 voxels. An expert annotator performed manual segmentations of proximal femoral and posterior iliac bones using in-phase (IP) Dixon MR images. Amongst four Dixon contrasts (IP, OP, PDFF, T2^*^), IP was deemed the most appropriate for manual annotation as it provided good soft tissue versus cortical bone contrast largely independent of marrow fat content and thus was most consistent among subjects (**Figure 1**). Expert annotations were only performed for the inferior (proximal femur) and superior (posterior iliac) sub-volumes. These annotations served as a reference standard in DL aided segmentation.

The original MF patient’s pelvic MRI volume did not provide a fixed number of images in each bone due to inconsistent patient positioning. The selection of sub-volumes for the pelvic bone sites therefore requires expert knowledge to determine the first and last image for each bone site. A single expert performed the systematic selection of the first and last slice of sub-volume to reduce the processing time. The expert used the most posterior point of the ilium as a reference, and the most superior slice and the most inferior slice of the segmentation were 1 cm and 3 cm from the reference point. For the femur, the top of the femur head was selected as the 1^st^ slice, and the last inferior slice was the last slice of the femur sub-volume. The MF patient’s proximal femur region extended across 14–32 images while posterior ilium spanned 11–14 images.

### 2.2 Segmentation workflow

**Figure 2:**
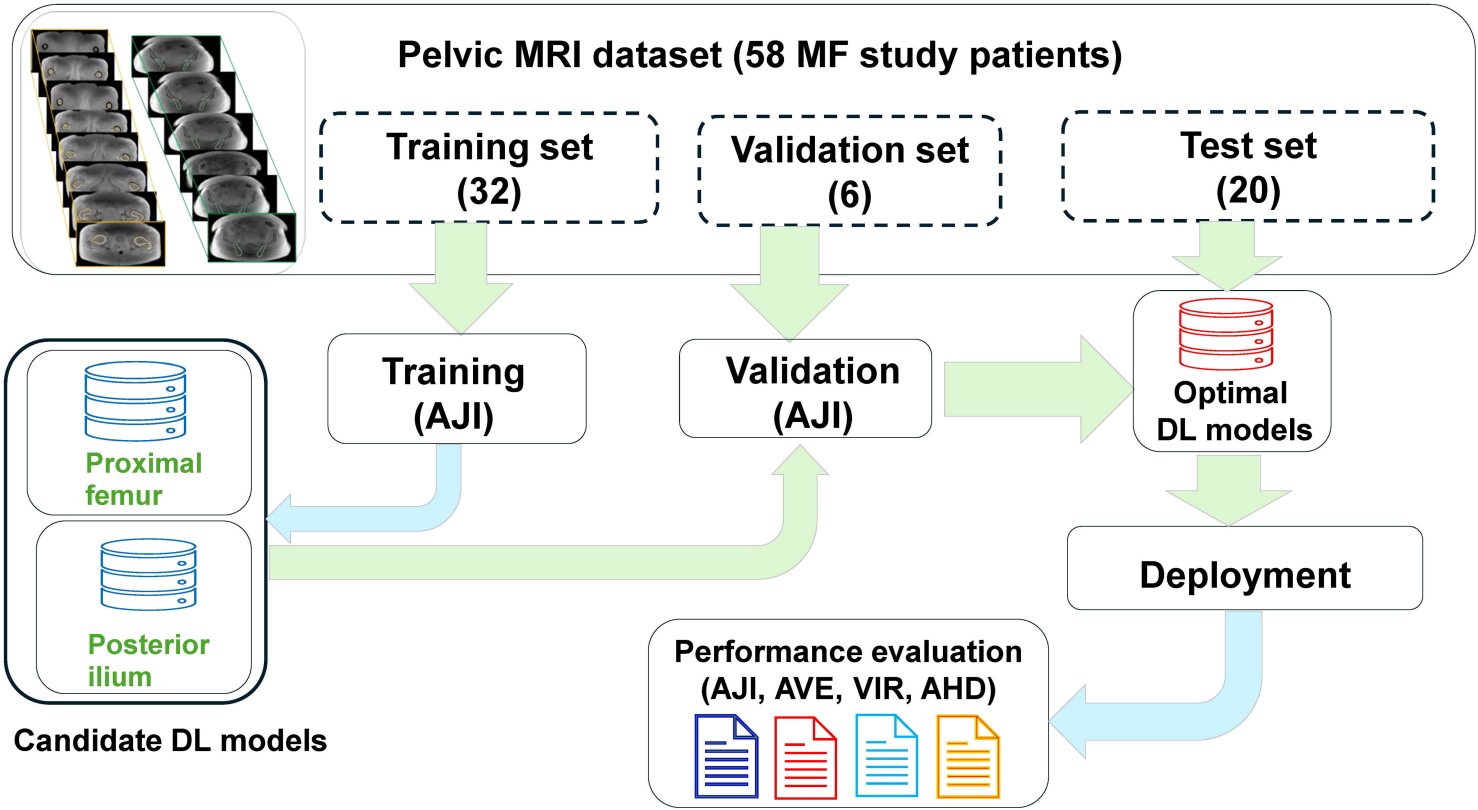
Deep learning (DL) segmentation optimization workflow. The training and validation sub-sets (with sizes are listed in parenthesis) were used for iterative model optimization at two bone sites (left workflow arm) based on average Jaccard Index (AJI) metrics. The optimal DL models were deployed for all sub-sets, including test. The segmentation performance was evaluated using volume difference and contour agreement metrics, including AJI, average Volume Error (AVE), Volume Intersection Ratio (VIR) and average Hausdorff distance (AHD), The top left inset shows example of expert annotations for femoral (left) and iliac (right) BM sub-volumes.

The BM segmentation model development and evaluation workflow is illustrated in **Figure 2**. The 58 MF patient MRI dataset was split into 55% (32/58) training, 10% (6/58) validation and 34% (20/58) test sub-sets respectively. Independent reviewer subjectively scored the fat content of the femoral and iliac bone marrow (BM) by inspecting PDFF maps. The training and validation subsets were selected by independent reviewer to include balanced samples of low to high BM fat content. The pelvic bone IP images and corresponding manual annotations were used iteratively to build separate U-Net models for segmentation of proximal femoral BM and posterior iliac BM. All models were developed, evaluated and tested on NVIDIA RTX A6000 GPU with 48 GB of memory and Pytorch library (version 2.3.0+cu118).

We compared segmentation performance of four model architectures (2D and 3D U-Net and attention (A)-U-Net) for each of the bone sites (**Figure 2**). The models were first trained by minimizing the average Jaccard index (AJI) loss to obtain all candidate DL models. The model hyperparameters were fine-tuned and the four best models were selected for each bone site using the validation dataset and maximizing AJI for optimization (**Supplementary Figures S1, S2**). The optimized models were then deployed to the held-out test set and the automated segmentations were compared to the reference annotations. Results of best model deployment on the test dataset (i.e. assessment metrics for agreement with manual annotations) were recorded and compared.

AJI metrics smoothness and stability were used for optimized model selection in DL-aided segmentation to aid generalizability and reliability (18, 19). Training and validation plots for four U-Net models in each bone (femur and ilium) along with brief description are provided in **Supplementary Figure S1** (2D U-Net) and **Supplementary Figure S2** (3D U-Net). 2D and 3D U-Net models differ from each other in terms of training time and hyperparameters, as summarized in **Supplementary Table S2**. Due to the difference between data premise of (3D) volume versus (2D) image input, U-Net models utilized distinct sample size, hyperparameters, weight updates (iterations) and training times. For 2D segmentation models, varying number of slices was allowed both for training and testing. To enforce consistent femoral sub-volume for 3D segmentation models, replication of the most inferior image with its associated mask was used as needed resulting in consistent femoral sub-volume of size 288x288x24 voxels. Superior iliac sub-volume needed inclusion of 2 to 3 existing iliac images resulted in consistent sub-volume size of 288x288x16 voxels for 3D models. The added replicate images were excluded from the downstream 3D model performance evaluation.

### 2.3 Post-processing

To improve segmentation and eliminate contouring errors for performance evaluation, post-processing was performed for hole filling and spurious noise removal after U-Net segmentation (e.g., **Supplementary Figure S3**). First, voxel discontinuities have been detected using nested contouring approach and seeded region growing was used to fill in the small holes. Selection of left and right bone regions have been made through connected component analysis and noise removal performed separately for left and right regions. The segmentations were visualized in 3D Slicer V4.11 and saved as meta-image header (MHD) format.

### 2.4 Performance evaluation metrics

Model performance was assessed on region-based and boundary-based overlap metrics. Region based overlap included average (over subjects) Jaccard Index (AJI), average Volume Intersection Ratio (VIR) and average Volume Error (AVE), while the boundary-based overlap was represented by average Hausdorff Distance (AHD). These metrics were chosen to ensure comprehensive performance analysis based on complementary measures of agreement (overlap and intersect) and bias (14, 18, 19). All metrics were calculated for individual slices of each patient MRI, and then averaged over the patients The inter-model variability for the performance metrics were assessed using standard deviation (SD). The F1-score was used to measure test’s accuracy (20, 21), similar to DICE coefficient (**Supplementary Materials**). The relevant performance metrics calculations and definitions are summarized below.

Jaccard Index: The Jaccard Index was defined as the ratio of the intersection of the predicted segmentation mask and the ground truth mask to their union. The Jaccard Index for a single slice or volume was calculated as


$$\;Jaccard\;Index=\;\frac{v_{r}\cap v_{p}}{v_{r}\cup v_{p}}$$

where $v_{r}$ and $v_{p}$ were the pixels/voxels of the segmentation mask drawn by expert as reference standard and predicted by U-Net, respectively. Additionally, the Jaccard index could be described using the concepts of true positives (TP), false positives (FP), and false negatives (FN):

True Positives (TP): The number of correctly predicted pixels/voxels of the target class.

False Positives (FP): The number of pixels/voxels incorrectly predicted as the target class.

False Negatives (FN): The number of pixels/voxels that were the target class but were not predicted as such.


$$Jaccard\;Index\;=\;\frac{TP}{FP+TP+FN}$$

Volume Intersection Ratio: The volume intersection ratio for the segmentation of a single scan was calculated as


$$Volume\;Intersection\;Ratio=\;\frac{v_{r}\cap v_{p}}{v_{r}}$$

where $v_{r}$ and $v_{p}\;$ were defined as above.

Volume Error: The VE quantifies the difference between reference and predicted mask volumes relative to the reference and is calculated as


$$\text{Volume Error }\left(\text{VE}\right)=100*\left(\frac{v_{r}-v_{p}}{v_{r}}\;\right)$$

where $v_{r}$ and $v_{p}\;$ are defined above. A positive value of volume error indicates under-segmentation (FN), and a negative value indicates over-segmentation (FP) by U-Net models.

Hausdorff Distance:

In our MRI analysis, each slice contains a left-right pair of segmentation (predicted, P, and reference, R) contours corresponding to the left and right bones on slice i:


$$L^{P}\left(i\right),L^{R}\left(i\right):the\;pair\;of\;set\;of\;contour\;points\;for\;left\;bone\;on\;slice\;i$$

$$R^{P}\left(i\right),R^{R}\left(i\right):the\;pair\;of\;set\;of\;contour\;points\;for\;right\;bone\;on\;slice\;i$$

Using our notation, the Hausdorff Distance is computed separately for each bone on every slice i . For left bone it is expressed as follows:


$$d_{H}^{L}\left(i\right)=\left\{\underset{p\in L^{P}\left(i\right)}{\max}\underset{r\in L^{R}\left(i\right)}{\text{min}}d\left(p,r\right),\;\underset{r\in L^{R}\left(i\right)}{\max}\underset{p\in L^{P}\left(i\right)}{\text{min}}d\left(p,r\right)\right\}$$

where d(p,r)  is the Euclidean distance between contour points p and r.

Similarly, for right bone Hausdorff distance becomes:


$$d_{H}^{R}\left(i\right)=\left\{\underset{p\in R^{P}\left(i\right)}{\max}\underset{r\in R^{R}\left(i\right)}{\text{min}}d\left(p,r\right),\;\underset{r\in R^{R}\left(i\right)}{\max}\underset{p\in R^{P}\left(i\right)}{\text{min}}d\left(p,r\right)\right\}$$

Since each slice provides both left and right contours, we define the representative Hausdorff distance for a slice as the maximum (worst-case) value between the two:


$$d_{H}^{max}\left(i\right)=\max\{d_{H}^{L}\left(i\right),\;d_{H}^{R}\left(i\right)\}$$

For a patient with N slices, the overall performance is summarized by two metrics:


$$Average\;Hausdorff\;Distance\;\left(AHD\right)=\frac{1}{N}\;\overset{N}{\underset{i=1}{\sum }}d_{H}^{max}\left(i\right)$$

$$\begin{array}{c} \text{Median Hausdorff Distance }\left(\text{MEDIAN}\_\text{AHD}\right)\\ =\text{median }\{d_{H}^{max}\left(i\right)\;|\;i=1,2,\ldots .N\}. \end{array}$$

### 2.5 Metric distribution analysis for model comparison

To assess the performance difference between 2D and 3D models, the pair-wise Wilcoxon rank sum test for metric comparison among AHD, AJI and AVE was performed. The performance metrics distributions were visualized using violin plots. All violin plots used kernel density estimation (KDE) for smoothing with lower bandwidth (0.3) to capture finer details (peaks and valleys) in test data results. The violin plots were generated in Python. Bonferroni correction was applied to adjust significant p-value thresholds for multiple-comparisons from initial significance level (α) 0.05 to the significant threshold p<0.003 for each U-Net model performance metric comparison test (22).

## 3 Results

The eight selected U-Net models reasonably mimicked the expert annotations from training data and generalized well on test data in both 2D and 3D for two bone-sites with good agreement VIR (90-96%) and AJI (85-88%) scores. Multi-facet evaluation of model performance on the validation sub-sets, from visual assessments to quantitative performance metrics and statistical details helped choose a preferred U-Net model for BM segmentation of the studied pelvic bone sites (proximal femur and posterior ilium). Additional details about models’ selection are included in the **Supplementary Materials**.

### 3.1 Qualitative segmentation evaluation

**Figure 3:**
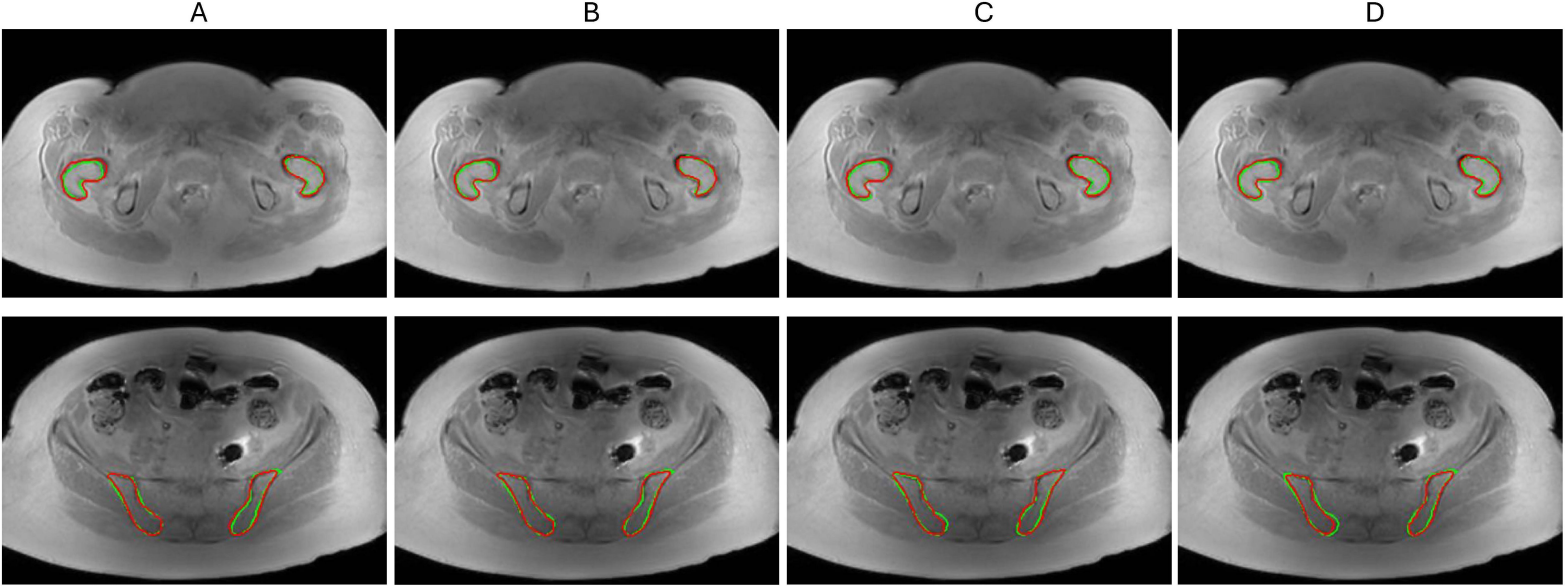
Segmentation model comparison for femoral BM (top) and iliac BM (bottom) on a test set **(A)** 2D U-Net **(B)** 2D A-U-Net **(C)** 3D U-Net **(D)** 3D A-U-Net. The reference contours are green and model segmentation contours are red.

An example of best selected models that adequately followed the reference contours and successfully localized the femoral and iliac BM is illustrated in **Figure 3**. The bone contours learned by U-Net models were largely consistent with reference outlines with minor variation around the boundaries. The model contours were slightly more deviant in femoral BM as compared to iliac BM where regional overlay fully covered the reference iliac contours except in 3D A-U-Net (**Figure 3D**, bottom).

**Figure 4:**
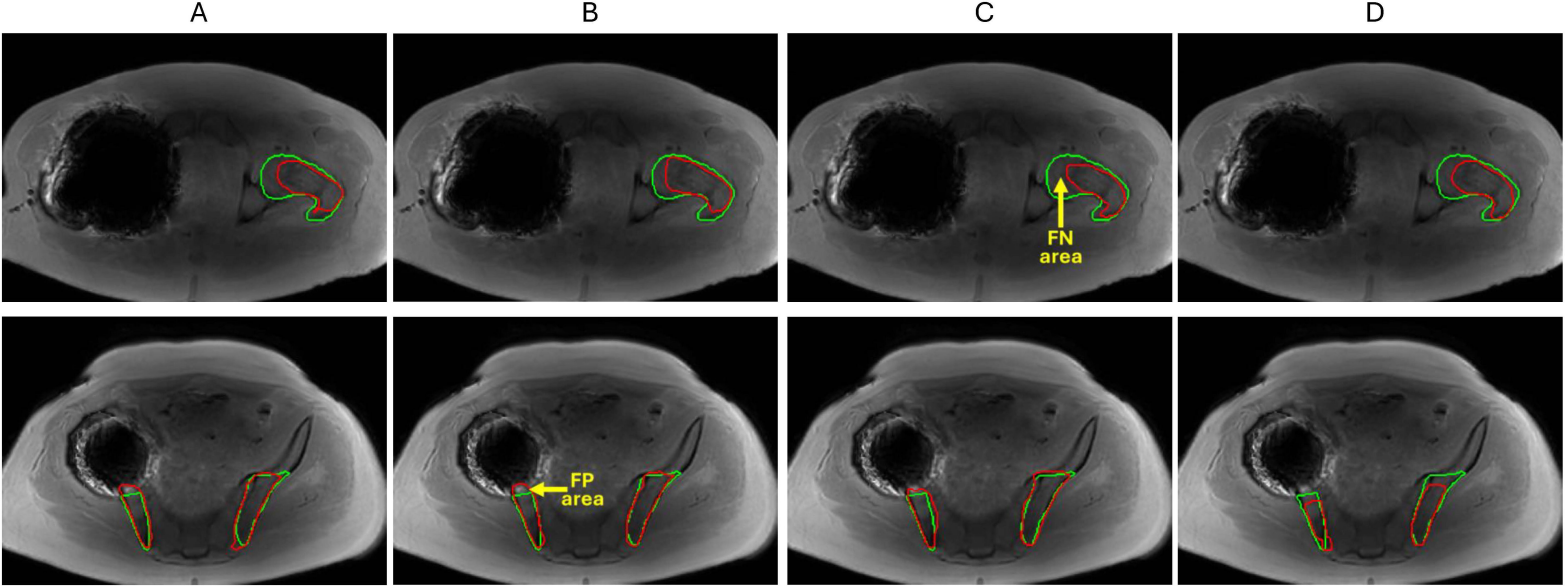
Segmentation model comparison for femoral BM (top) and iliac BM (bottom) for a challenging test patient (with hip implant artifact) **(A)** 2D U-Net **(B)** 2D A-U-Net **(C)** 3D U-Net **(D)** 3D A-U-Net. The reference contours are green and model segmentation contours are red. Yellow arrows point at examples of false negative (FN) and false positive (FP) areas.

An example of large segmentation errors observed for the studied data set is shown in **Figure 4** for a single test MF patient with challenging metal artifact due to a hip implant. Femoral bone (top) BM segmentations in **Figure 4C** follow reference contours relatively well but also miss some true BM pixels causing false negatives (FNs). The iliac bone (bottom) segmentations by all U-Net models also largely overlap with expert annotations but incur few false positives (FPs) outside the reference boundaries. This example showed that U-Net models largely trained on symmetric bones exhibited the ability to generalize well for a single-side bone segmentation. Additional examples of segmentation errors observed primarily for the marginal slices of imaged bone sub-volume are illustrated in **Supplementary Figure S3**. We also observed that denoising post-processing affected less than 4% of total segmentation volumes and was apparently required more often for 3D than for 2D models and for femur versus ilium, likely reflecting limited training set size for the bone site anatomy.

### 3.2 Quantitative performance evaluation

**Tables 1** and **2** systematically compare region and contour agreement and error scores (AJI, AVE, VIR, AHD and MEDIAN_AHD) for proximal femur and posterior iliac BM segmentation across training, validation and test sets after denoising post-processing. This analysis confirms the moderately better performance of 2D U-Net models over 3D U-Net models which otherwise were not obvious from visual inspection of segmentations. As expected, validation and testing performances were always lower than the corresponding training performances in all metrics, except femur AVE and AHD.

**Table 1:** Performance metrics [mean ± standard deviation (SD)] for proximal femur segmentation with different tested U-Net models.

Bone-Site	Proximal Femur					
DL Models	Dataset	AJI%	VIR %	AVE%	AHD (mm)	MEDIAN_AHD (mm)
2D U-Net	Training	89.1 ± 2.0	94.3 ± 1.5	0.7 ± 5.5	29.7 ± 7.1	5.1
	Validation	88.5 ± 1.8	93.6 ± 2.3	0.6 ± 8.0	31.4 ± 5.3	5.6
	**Test**	**85.5 ± 6.7**	**93.3 ± 2.2**	**0.2 ± 8.0**	**28.4 ± 8.9**	**5.7**
2D A-U-Net	Training	89.0 ± 1.4	92.5 ± 1.6	6.0 ± 5.0	28.5 ± 6.5	5.4
	Validation	88.3 ± 2.2	92.2 ± 3.0	5.0 ± 9.0	32.0 ± 3.7	5.1
	**Test**	**86.9 ± 2.5**	**91.5 ± 3.5**	**5.0 ± 9.0**	**26.8 ± 9.3**	**6.2**
3D U-Net	Training	87.8 ± 2.0	93.0 ± 2.0	2.4 ± 6.4	26.7 ± 6.3	5.8
	Validation	86.0 ± 3.0	91.3 ± 3.0	2.9 ± 10.0	28.3 ± 6.9	6.8
	**Test**	**85.7 ± 2.3**	**91.0 ± 3.1**	**5.2 ± 10.2**	**25.5 ± 9.0**	**6.2**
3D A-U-Net	Training	87.8 ± 2.1	91.6 ± 2.1	7.3 ± 6.4	26.9 ± 7.0	5.6
	Validation	86.1 ± 3.0	89.6 ± 3.4	10.3 ± 7.7	29.7 ± 5.7	6.7
	**Test**	**84.9 ± 4.0**	**89.0 ± 5.0**	**11.6 ± 10.8**	**25.8 ± 9.5**	**5.8**

The values in bold indicate performance metrics for the independent test set.

**Table 2:** Performance metrics [mean ± standard deviation (SD)] for posterior ilium segmentation with different tested U-Net models.

Bone-Site	Posterior Ilium					
DL Models	Dataset	AJI%	VIR %	AVE%	AHD (mm)	MEDIAN_AHD (mm)
2D U-Net	Training	90.3 ± 1.5	96.4 ± 1.1	-4.5 ± 5.2	9.6 ± 4.0	5.1
	Validation	89.4 ± 1.1	96.5 ± 1.7	-7.3 ± 7.4	10.1 ± 3.0	5.5
	**Test**	**87.0 ± 3.6**	**95.7 ± 2.6**	**-10.4 ± 11.5**	**18.1 ± 10.7**	**6.8**
2D A-U-Net	Training	91.0 ± 1.5	96.0 ± 1.3	-1.5 ± 5.0	8.9 ± 2.6	4.4
	Validation	89.4 ± 1.2	95.6 ± 1.9	-4.6 ± 5.6	15.0 ± 6.8	5.6
	**Test**	**88.0 ± 2.4**	**95.5 ± 1.8**	**-7.5 ± 9.0**	**16.1 ± 8.2**	**5.7**
3D U-Net	Training	88.0 ± 1.8	93.4 ± 2.4	-0.2 ± 6.6	12.7 ± 4.0	6.3
	Validation	86.8 ± 2.0	94.0 ± 2.8	-3.8 ± 7.4	18.9 ± 11.1	8.9
	**Test**	**85.0 ± 5.6**	**93.0 ± 6.0**	**-4.3 ± 12.0**	**20 ± 13.0**	**8.4**
3D A-U-Net	Training	87.8 ± 2.1	91.5 ± 3.2	6.0 ± 6.8	14.3 ± 6.3	6.9
	Validation	87.6 ± 1.9	92.3 ± 2.7	2.7 ± 6.5	16.1 ± 7.5	6.1
	**Test**	**85.1 ± 4.7**	**90.6 ± 5.3**	**3.5 ± 10.7**	**19.1 ± 10.3**	**6.8**

The values in bold indicate performance metrics for the independent test set.

**Table 1** illustrates that the slice-wise 2D AHD for all four U-Net models was relatively high in femoral BM (26–32 mm) with wide range of standard deviations (SD=4-9 mm). Median AHD for test set varied less between 2D and 3D U-Net models (5.7-6.2 mm) indicating comparable performance for directed boundary distance in femur. This confirmed the likely source of higher 2D AHD values from marginal slices in the femur volume. In femoral BM, both AVE and SD(AVE) were lower for 2D versus 3D model segmentations. e.g. (AVE: 0.2-6% versus 2-12% with SD 5 – 9% versus 6-11%). Interestingly, the mean values of contour-distance (AHD) decreased for the test set as compared to training set for 2D models (e.g. from 27-28 mm versus 29–30 mm). The AHD for 3D U-Net models (26 mm) were slightly lower than for 2D models and similarly lower than training AHD (27 mm) and similar SD (9-10 mm).

**Table 1** further shows that 2D U-Net achieved marginally higher VIR on the test set compared to the 2D A-U-Net in proximal femur (93% versus 92%) while the trend was reverse for AJI (86% versus 87%). The 2D models agreement metrics (AJI, VIR) reflected similar performance across training, validation and test sets (AJI: 86-89%, VIR: 92-94%), consistently better than for 3D models (AJI: 85-88%, VIR: 89-93%). The relatively small standard deviations (SDs) of 2-3% were observed for 2D A-U-Net and 3D U-Net but roughly doubled from training to test set for agreement measures of 2D U-Net and 3D A-U-Net. Improvement in agreement and lower variability between 2D and 3D models for training, test and validation set demonstrated that 2D models marginally outperformed the 3D models for femur segmentation.

**Table 2** summarizes the performance of four iliac U-Net models using overlap and boundary metrics. The ilium contour errors are surprisingly lower in comparison to femur (2D Ilium AHD: 9-18 mm, 2D femur AHD: 26-32 mm). Contrary to femur, no anomalous trends on test versus training set were observed in AHD of iliac U-Net models with both mean (16–20 mm) and SDs (8–11 mm) increasing for the test set. **Table 2** shows that 2D U-Net and 2D A-U-Net training, validation and testing for iliac segmentation models were consistent in both AJI and VIR and small performance decline (3-5%) was observed from training to validation and test (test AJI: 87–88%, test VIR: 95-96%). Test 2D segmentation errors for U-Net and A-U-Net in iliac bone site ranged (AVE: 7–10%, AHD: 16–18 mm). The 3D iliac models also attained higher AJI (85– 88%) and differed from each other only in VIR by 3% (e.g. test VIR: 90% versus 93%). Comparison of 2D versus 3D posterior ilium segmentations from **Table 2**, shows that the 3D training, validation and test accuracy were also higher for 2D iliac models (2D AJI: 87–91% versus 3D AJI: 85–88%, 2D VIR: 95–97%, versus 3D VIR: 91–94%).

**Tables 1** and **2** allow comparison between the two BM bone-sites. In both bone-sites, the performance of 2D U-Net and 2D A-U-Net were satisfactory and close (2D AJI: 87-91%, 2D VIR: 91-96%). Differences in agreement accuracy between bones indicated more consistent performance in ilium (e.g. 2D iliac VIR: 95–96% versus 2D femur VIR 91-94%). Finally, comparing the 3D model segmentation performance between **Tables 1** and **2** to analyze bone-site impact on 3D training, validation and testing data revealed that the AJI was similar for 3D U-Net and 3D A-U-Net in both femur and ilium (3D AJI: 85-88%). 3D models VIR performance declined more in femur as compared to ilium (4% versus 2% decline).

Except for 3D A-U-Net, all ilium segmentation models had a negative AVE bias indicating tendency to over-estimate volume compared to expert segmentations. In contrast, femur model segmentation AVE bias was largely positive consistent with the tendency to underestimate BM volume. The volume contour error (MEDIAN_AHD) for all four U-Net models attained consistent range in both pelvic bones (femur: 5–7 mm, ilium: 5-9 mm). Overall higher AVE-SD and AHD variability in 3D models further confirmed lower performance of 3D models compared to 2D U-Net and 2D A-U-Net (2D AVE SD: 2.5% versus 3D AVE SD: 3.5%, 2D AHD SD: 3–11 mm versus 3D AHD SD: 5-13 mm).

### 3.3 Metric distribution analysis

**Figure 5:**
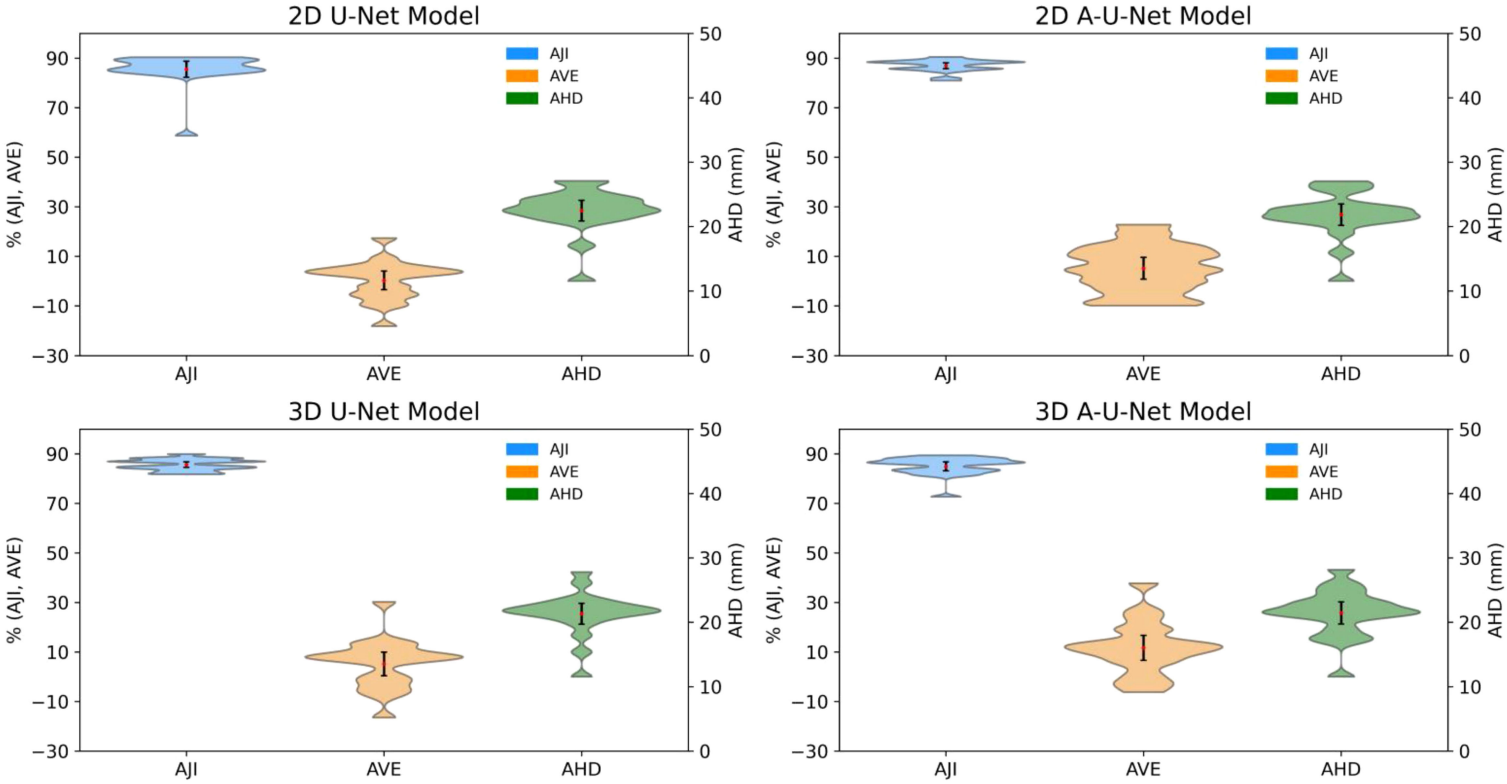
Distribution of AJI, AVE and AHD for femoral segmentation test data. Black error bars represent one standard deviation (SD) from the mean while colored triangles correspond to data ranges. The metric distribution is color-coded in the legend. The left axis is shared for AJI and AVE (%), while right axis is for AHD (mm).

**Figure 6:**
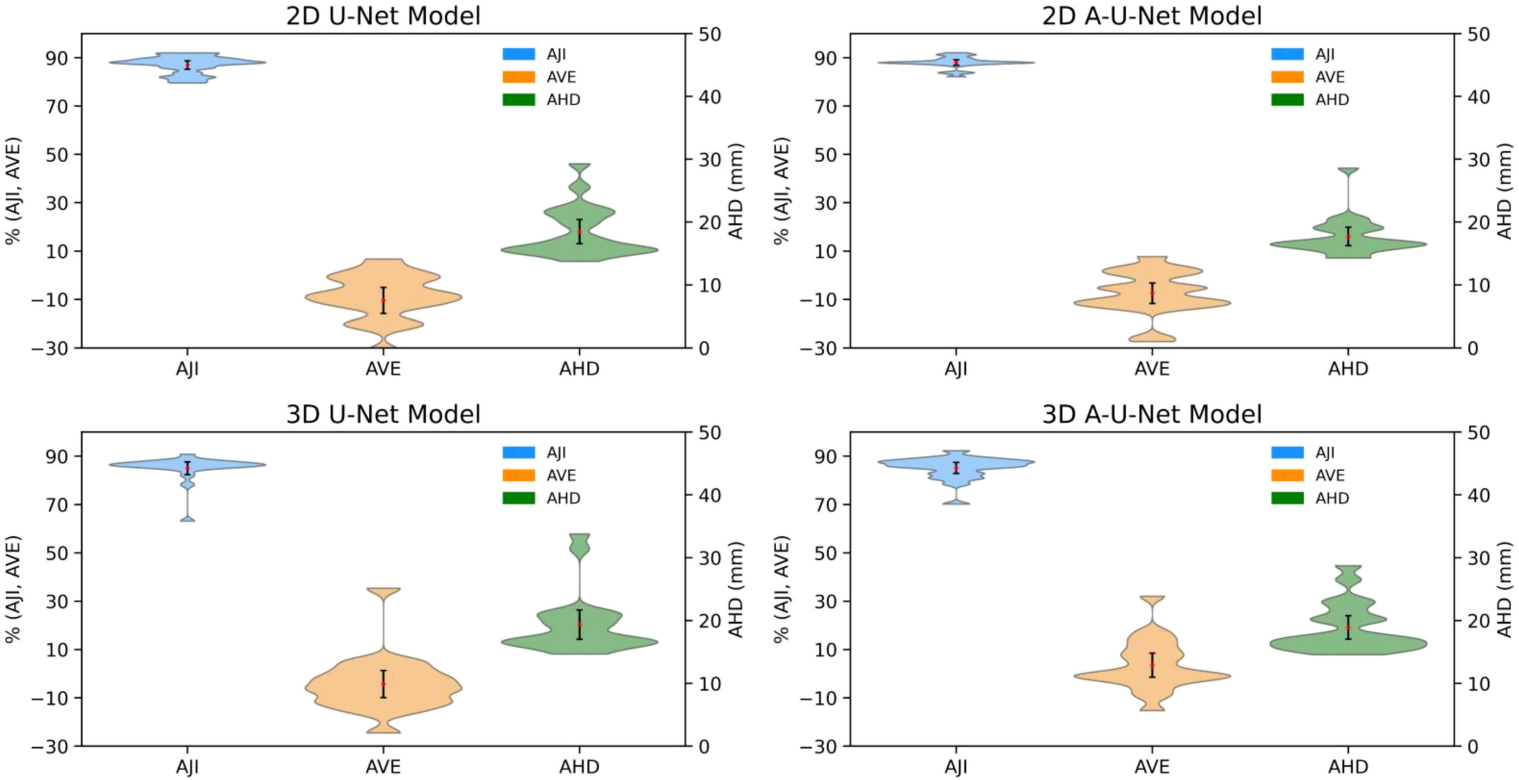
Distribution of AJI, AVE and AHD for iliac segmentation test data. Black error bars represent one standard deviation (SD) from the mean while colored triangles correspond to data ranges. The metric distribution is color-coded in the legend. The left axis is shared for AJI and AVE (%), while right axis is for AHD (mm).

**Figures 5** and **6** for femur and ilium provided further insight into the distribution shape, mean, and range of three most error-sensitive performance metrics (AJI, AVE and AHD) for the test data with all four selected U-Net models in each bone site.

The distribution’s shape and width of violin plots revealed subtle differences in variability and performance consistency, particularly between 2D and 3D models and between U-Net and A-U-Net configurations. Multiple distribution modes were resolved for 3D and 2D model performance metrics for femur, likely reflecting the higher susceptibility for error at the bone margins due to inconsistencies of image volumes.

The comparison of AJI violin plots of femoral bones (**Figure 5**) and iliac bones (**Figure 6**) indicated uniformly high performance with maximum density around 90% for all four U-Net models in both bone-sites. Femoral bone metric distributions (**Figure 5**) showed comparable performance for 2D versus 3D models with marginally narrower distributions (less variability for 2D A-U-Net AJI (range: [85%,95%]). Most of 2D U-Net and A-U-Net AJI had clustered around 86% with just 2-5% narrow error bars. The femur metric distributions are complex and multi-modal likely indicating limitations of small test data set.

The low mean errors for both 2D and 3D U-Net models for AVE demonstrated generally strong agreement with reference volume. Both 2D models performed better than 3D with respect to AVE (2D femoral AVE range: [-20, 20], 2D ilium AVE SD: [-30, 10] versus 3D femoral AVE range: [-20, 35], 3D ilium range: [-25, 30]). Iliac bone metric distributions (**Figure 6**) followed the similar trends of higher AJI and lower AVE SD with notably narrower AHD, particularly for 2D A-U-Net model.

All four U-Net models AHD distribution in for femur versus ilium (**Figures 5**, **6**) exhibited consistent trend of high data density at higher AHD values (> 20 mm) for femur and at lower AHD (< 18 mm) for ilium, confirming better performance for ilium than femur. The mean values being higher than the maximum width pointed out the presence of outliers, likely for the bone margins.

**Figure 7:**
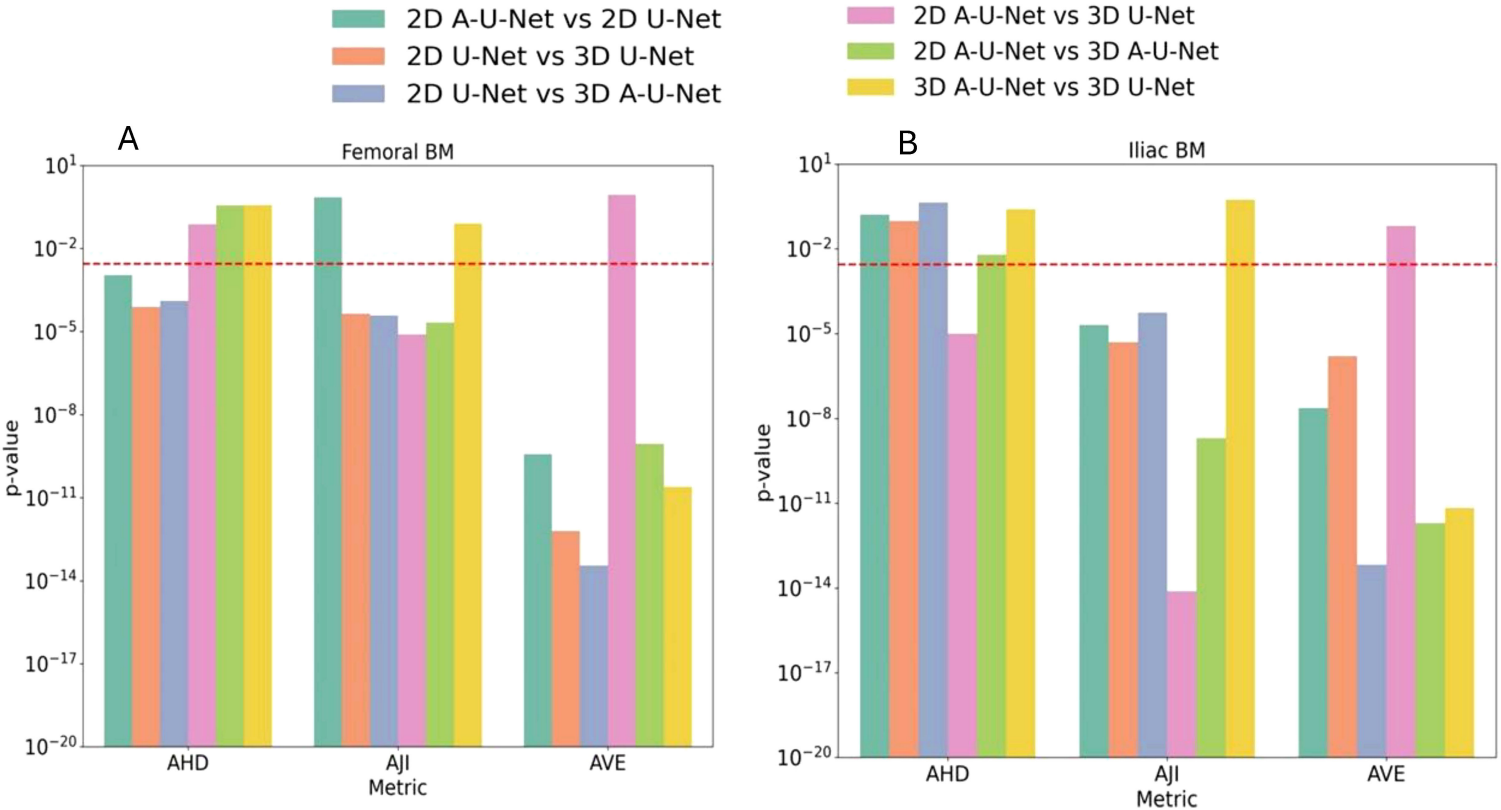
**(A, B)** p-values of pairwise comparison of AHD, AJI and AVE for UNET models (color-coded in the legend) for segmentation performance on a test dataset. A red dashed line in p-value plots represented the Bonferroni-corrected significance threshold p<0.003.

**Figure 7** depicts the results of Wilcoxon rank sum test in the form of grouped bar plot for p-values in femoral bone and iliac BM respectively. Each metric group thus consisted of six bars representing the six unique comparisons, with a total of 18 comparisons for the three metric groups. Notably, non-significant p-values above the threshold were observed for 6 comparisons in femur and 7 in iliac segmentation models. The AJI and AVE metrics were most sensitive to the differences between the models There was no significant difference in 3D A-U-Net versus U-Net performance for iliac and femoral BM segmentation for AJI and AHD. Ilium models also showed that 3D U-Net and 3D A-U-Net are not significantly different. Except for AHD, the differences between 2D and 3D models were significant. Combined with absolute performance metric values, the significant differences between other models helped identify the overall most effective model for MF bone marrow segmentation as 2D A-U-Net.

**Supplementary Table S3** indicated that the proximal femur and posterior ilium (U-Net and A-U-Net) 2D models exhibited stronger performance with F1 scores ranging between 89% and 90%. Close F1-scores for 2D models (~2% difference), indicated that these models achieved a similar balance between precision and recall in each bone-site. This suggested consistent accuracy and reliability in BM segmentation. Iliac 2D U-Nets had lower SD (0.02-0.03) suggesting more consistent performance across different test runs than for femoral bone. In comparison to 2D, the 3D models showed lower F1-score in both bones (Femur: 87% versus 89%, Ilium 87% versus 90%).

Overall, compared to 3D segmentation models, clear performance enhancement was observed for 2D A-U-Net in ilium, while 2D models in femur showed better agreement (AJI, VIR, F1, AVE-SD) but slightly higher contouring errors (AHD) and lower precision (**Supplementary Table S3**).

**Supplementary Figure S4** illustrates Bland-Altman agreement analysis for mean fat fraction (FF) for expert versus preferred DL segmentation model (2D A-U-NET) for the test set. This analysis confirms good agreement (LOA<3%) between FF quantifications for expert versus model segmentations.

## 4 Discussion

The focus of our study was to find the best U-Net models to automate the BM segmentation in two pelvic bones (proximal femur and posterior ilium) for MF patient Dixon MRI. We developed, validated and compared four distinct U-Net models based on 2D and 3D U-Net and A-U-Net to identify superior models for each pelvic bone site. All eight independently validated U-Net segmentation models in our study showed strong agreement with reference BM segmentations. Overall, the optimized segmentation models in each bone achieved adequate performance and overlap accuracy of boundary delineation for complex and granular segmentation tasks on training, validation and test sets. Our 2D models had higher F1-scores compared to the 3D models (2D: 89-90% versus 3D: 86-88%), good AJI (2D: 87-88% versus 3D: 85%-86%) and excellent VIR (2D: 96% versus 3D: 89-93%). All models demonstrated low segmentation errors and consistently reproduced the reference BM volumes (AVE bias between -11% and 12%). Ilium models were predominantly under-segmenting, while femur models were prone to over-segmentation. Higher variability was observed for 3D AVE versus 2D AVE at both bone sites. Our BM segmentation results suggested that the 2D A-U-Net performed better on average and provided more reliable predictions for both pelvic bone segmentation where the training set contained variable coverage of the femoral shaft for MF patients.

Interestingly, 3D segmentations exhibited comparable performance versus 2D in boundary match (AHD) between models and reference for femur but markedly higher deviations and variability for ilium. The statistical evaluation of femoral and pelvic models reflected that 2D models were more robust than their peer 3D models. Our study further found that AJI was the most stable metric to compare alternative 2D and 3D U-Net BM segmentation models for both bone-sites. Moreover, femoral segmentation showed greater absolute contour mismatches (AHD=26-28 mm) than iliac model segmentations ((AHD=16-20 mm)) reflecting that different bones require training distinct segmentation models. In other words, a DL model trained on a single bone-site would not optimally segment every bone. In our case, the iliac BM voxels occupied larger area with more diverse orientations as compared to femoral BM voxels on individual images but had smaller scanned sub volume (400 versus 735 training images) which is a likely reason for different performance of U-Net models on different bones.

Our best 2D A-U-Net (femur and iliac) segmentation model performed comparable to 3D U-Net lumbar vertebra segmentation model described in the recent quantitative PDFF MRI study (11). The vertebral segmentation study involved 30 healthy training and 12 testing subjects, which were comparable to our training and testing sets (training:32, testing:20). Our validation set AJI achieved substantial gain in both pelvic bones (femur and ilium) with an approximate increase of 8% to 10% compared to reported lumbar vertebral AJI. Our mean F1-score on the test set were remarkable close to the cited vertebral F1-score (dice similarity) of 86-90%. The precision-recall trend in pelvic bones and vertebral bones also looked similar. However, the vertebral U-Net model training configuration (hyperparameters) and validation methodology did not include tuning and optimization. Our optimized 2D U-Net models also outperformed another Dixon lumbar vertebra segmentation study on two test sets (annotated by different observers) in both AJI and dice similarity with substantial 10% advancement in AJI and 5% increase in F1-score (13). In addition to different U-Net training and optimization approaches, a probable reason for difference in observed segmentation performance could be that vertebral bodies inter slice spatial locations on sagittal images were subject to change while both femur and iliac spatial coordinates remained consistent in all images. Importantly, the achieved agreement with the manually annotated reference for the test set in our study exceeds the reported inter-observer agreement by 15% (13).

Our present study confirmed U-Net models’ potential for accurate BM segmentation as found in previous studies. Furthermore, it revealed the utility of IP image training for better model generalization for BM diseases with variable fat content for patient population like MF. Practical considerations about DL aided segmentation workflow optimization for both 2D and 3D U-Net models along with comprehensive segmentation performance evaluation and statistical analysis will also benefit other quantitative MRI studies of bone marrow disease. In addition to automated segmentation, the insights gained by our study include separate U-Net model utility for individual bone sites, empirical evidence of 2D U-Net preference over 3D U-Net for varying patient positioning with small, annotated training set, substantial difference in overlap and boundary performance metrics and practical workflow to find single best DL model for each pelvic bone-site. This segmentation automation will save about 20 minutes of expert time per subject/time point and will likely improve repeatability and remove expert-dependent bias (11, 12). Our previous study in murine model of MF using similar DL segmentation models indicated significantly higher inter-observer reproducibility and test-retest repeatability by automated segmentation (12).

Our study had several limitations. First, this was a single center rare disease (MF patient) study in specific bone-sites (proximal femur and posterior ilium) that may limit the segmentation model’s applicability (22, 23). However, this study has the largest patient cohort reported to date for MF rare disease and proposed use of IP images for segmentation, makes this approach less dependent on relative BM fat content. Second, all training, validation and test subsets were acquired on a single scanner with uniform scan protocol which further limits the direct generalization of the models for different acquisition protocols. Small datasets with limited and biased annotations are prevalent in imaging research (24). Our study also utilized a small MF data set with a single expert annotation and did not assess inter-observer reproducibility. Therefore, manual quality check for independent test sets, multi-reader studies and correction of new cases would be required for comprehensive evaluation. Alternatively, model retraining and advancement in segmentation workflow automation would be an option to further reduce the manual annotation efforts. Another limitation was the absence of segmentation ground truth which was partially resolved by training U-Net models (25). Single expert delineations were considered reliable as a reference for our application, although they might not capture all relevant details due to human perception limitations. Even repeated annotations from the same expert may be inconsistent and might limit DL model performance. Furthermore, expert intervention would be required to manually define sub-volumes for the model application to new data at femur and ilium sites, which limits full workflow automation. This step can be streamlined in future applications. Another important technical issue identified during this study was inconsistency of patients positioning during MRI acquisition, resulting in image volume variability affecting the 3D U-Net model performance (26). By their nature, 2D U-Net segmentation models are less sensitive to patient positioning and thus would be preferred for pelvic BM MRI studies.

Future work will apply the best developed models to advance segmentation automation in the ongoing MF studies by leveraging limiting annotated data and extensive un-annotated data. The best DL models will be used to generate pseudo labels for expert review and adjustment. The combined annotated and pseudo-labeled data can be used iteratively to retrain the model, continuously enhancing its accuracy and reliability. This semi-supervised setting may outperform traditional transfer learning and, in some cases, self-supervision. It will also reduce dependency on extensive manual labeling to accelerate the segmentation efficiency and throughput (27, 28). The derived segmentations will be applied to quantitative PDFF maps to measure longitudinal changes in MF bone marrow. We also plan to use these segmentations for transfer to other quantitative MRI contrasts (e.g., ADC) at multiple imaging time points (currently 5–7 time points per patient). The improved automation in segmentation workflow is vital for timely development, validation and implementation of quantitative biomarkers and design therapeutics strategies in MF patients’ treatment response.

## 5 Conclusion

This study developed, validated and tested independent U-Net models to effectively segment BM in quantitative PDFF MRI (IP volumes) of MF patients. The best selected models automatically detected, and segmented BM in pelvic bones (proximal femur and posterior ilium) and showed strong agreement with reference BM segmentations. The comparative analysis revealed that 2D U-Net and 2D A-U-Net out-performed their peer 3D models for BM segmentation in patients’ target pelvic bones. 2D A-U-Net model performance was more robust in comparison to other DL models in both femur and ilium. Overall, selected U-Net models demonstrated promising performance to accelerate segmentation and accurately localize BM regions for future patient studies. The developed BM segmentation models lay the foundation to assess heterogeneity and spatial distribution of the PDFF in pelvic bones which enhances QIB precision. The U-Net based segmentation automates the bone marrow delineation with improved accuracy to facilitate future clinical adoption of MRI for MF patients.

## Data Availability

The raw data supporting the conclusions of this article will be made available by the authors, without undue reservation.
