# Supplementary material for: A deep learning aided bone marrow segmentation of quantitative fat MRI for myelofibrosis patients
Source: Front Oncol. 2025 May 23;15:1498832. doi: 10.3389/fonc.2025.1498832 (PMC12140989; doi:10.3389/fonc.2025.1498832)
Supplement: Supplementary file 1 [file DataSheet1.pdf]

Supplementary Material

1 Supplementary Data

Supplementary Table S1 provides the patient demographics and image counts for each bone site.

1.1 Training hyperparameters

Supplementary Table S2 summarized two types of experimental conditions for DL-aided BM segmentation. The first category comprises machine specific requirements including GPU, Pytorch library and maximum time to train four DL-models: 2D U-Net, 2D A-U-NET, 3D U-Net, and 3D A-U-Net. The second category summarizes training hyper-parameters including final epoch numbers for optimized models. All U-Net models were trained using a learning rate of 0.001, Adam optimizer and soft Jaccard loss for total 100 epochs. Additional tuning was performed using validation set Jaccard. The architectural difference between 2D and 3D UNETs along-with sub-volumes sizes of femoral section (288 x 288 x 24) and iliac section (288 x 288 x16), led to different training times for both bone sites. The proximal femur model took a maximum ~ 4 hours to train while posterior iliac model training time was approximately half of that. Due to the differences between 2D versus 3D datasets, training the 2D UNET involved batch size of 20 to 40 images while in 3D it was 5 to 8 image volumes for the two bone-sites. Batch size limits would vary for different GPU cache sizes and impact the frequency of weight updates by splitting the training dataset into batches. Table S2 shows that the maximum batch size (B) supported by GPU and total number of training samples (N) together determine the number of batches (b) processed by GPU in a single epoch (b ~N/B). The product of (b) and the maximum number of epochs (E) yields the total number of iterations representing the frequency of weight updates during the complete training (f =b*E).

1.2 Training and validation performance trend

Training and validation Average Jaccard Index (AJI) are plotted versus 100 epochs for 2D U-Net models in Figure S1. The best model was selected for an epoch where the model had high and stable AJI on the validation set. Figure S2 shows training and validation AJI metrics for 3D U-Net models plotted for 100 epochs. The AJI for 3D U-Net models also exhibited smoothness but 3D learning was notably less stable (i.e. noisier) than 2D indicated as evident from less stable 3D AJI vs Epoch plots.

1.3 Summary of segmentation errors

Figure S3 exemplifies the possible false positive (FP) and false negative (FN) predictions from several patients in test set by 2D U-Net, 2D A-U-Net, 3D U-Net and 3D A-U-Net. It has been observed that femoral bone segmentations posed more challenges for U-Net models possibly due to variation in bone shape. The sporadic noise islands appearing on individual slices away from bone volumes were removed by denoising post-processing.

1.4 Precision, recall and F1 score performance evaluation metrics

The precision, recall, and F1-score are defined as follows:

$$precision = \frac{TP}{TP + FP}$$

$$recall = \frac{TP}{TP + FN}$$

$$F1\ score = \frac{2 * precision * recall}{precision + recall}$$

where True Positives (TP) is the number of correctly predicted pixels/voxels of the target class, False Positives (FP) is the number of pixels/voxels incorrectly predicted as the target class, and False Negatives (FN) is the number of pixels/voxels that were the target class but were not predicted as such.

The F1 score is the harmonic mean of precision and recall, providing a single balanced metric. Dice similarity coefficient is mathematically equivalent to the F1-score when applied to binary segmentation tasks. Table S3 summarizes the F1 similarity scores for the segmentation models in the test sub-set.

Figure S4 illustrates Bland-Altman agreement analysis for mean fat fraction for expert versus best DL segmentation model (2D A-U-NET) for the test sub-set. This analysis confirms good agreement (LOA<3%) between FF quantifications that use these segmentations.

2 Supplementary Figures and Tables

2.1 Supplementary Tables

Table S1. Patient demographics and image counts for data subsets

Data unit	Patients	age (yrs)	Gender	Full volume images	2D images	
Dataset	Count	median [range]	female: male	Count	Proximal femur	Posterior ilium
Training	32	63[29,88]	16:16	1824	735	400
Validation	6	59[52,68]	4 : '2	342	137	75
Test	20	64[39,82]	13 : '7	1140	419	253

Table S2. Training time and hyperparameters for DL-aided segmentation. **All models were developed, evaluated and tested on NVIDIA RTX A6000 GPU with 48 GB of memory and Pytorch library (version 2.3.0+cu118).**

DL-model training time	DL-model	Training hyperparameters				
		Best model selection (epochs)	Batch size	Sample size	Batches per epoch	updates in 100 epochs
			B	N	b ~N/B	f
Proximal femur Time ~ 4 Hours	2D UNET	16	40	735	~19	1900
	2D A-UNET	17				
	3D UNET	46	5	32	~7	700
	3D A-UNET	44				
Posterior ilium Time ~ 3 Hours	2D UNET	20	40	400	10	1000
	2D A-UNET	26				
	3D UNET	56	8	32	4	400
	3D A-UNET	56				

Table S3 Precision, recall and F1-score of four selected U-Net models in femur and iliac bones. The bold reflect the maximum mean ± std in each column and bone section.

Bone-site	DL-Models	% Precision	% Recall	% F1-Score
**Proximal Femur**	**2D UNET**	90.2 ± 0.09	**89.2 ± 0.07**	89.2 ± 0.07
	**2D A-UNET**	**93.0 ± 0.09**	87.0 ± 0.08	**89.2 ± 0.07**
	**3D UNET**	91.1 ± 0.09	85.7 ± 0.07	87.6 ± 0.06
	**3D A-UNET**	**93.1 ± 0.08**	82.6 ± 0.09	86.6 ± 0.07
**Posterior Ilium**	**2D UNET**	86.0 ± 0.06	**93.6 ± 0.04**	89.3 ± 0.03
	**2D A-UNET**	**88.0 ± 0.05**	93.0 ± 0.04	**90.0 ± 0.02**
	**3D UNET**	86.0 ± 0.05	88.3 ± 0.10	86.5 ± 0.06
	**3D A-UNET**	**90.0 ± 0.03**	85.1 ± 0.08	87.0 ± 0.05

2.2 Supplementary Figures

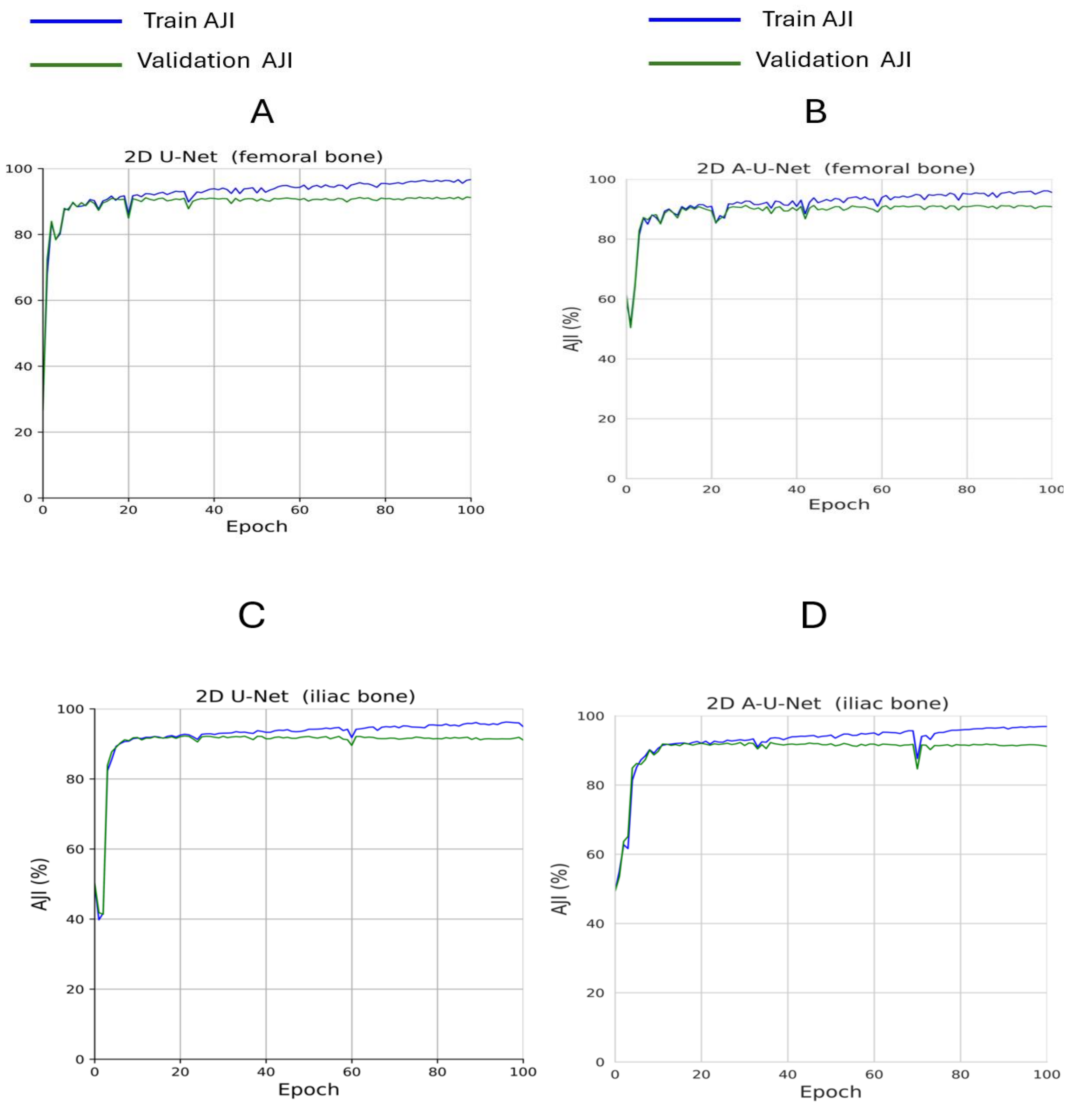

Figure S1. Training and validation performance trends (color-coded in the legend) for 2D U-Net best models (A) femoral bone (2D U-Net) (B) femoral bone (2D A-U-Net) (C) iliac bone (2D U-Net) (D) iliac bone (2D A-U-NET)

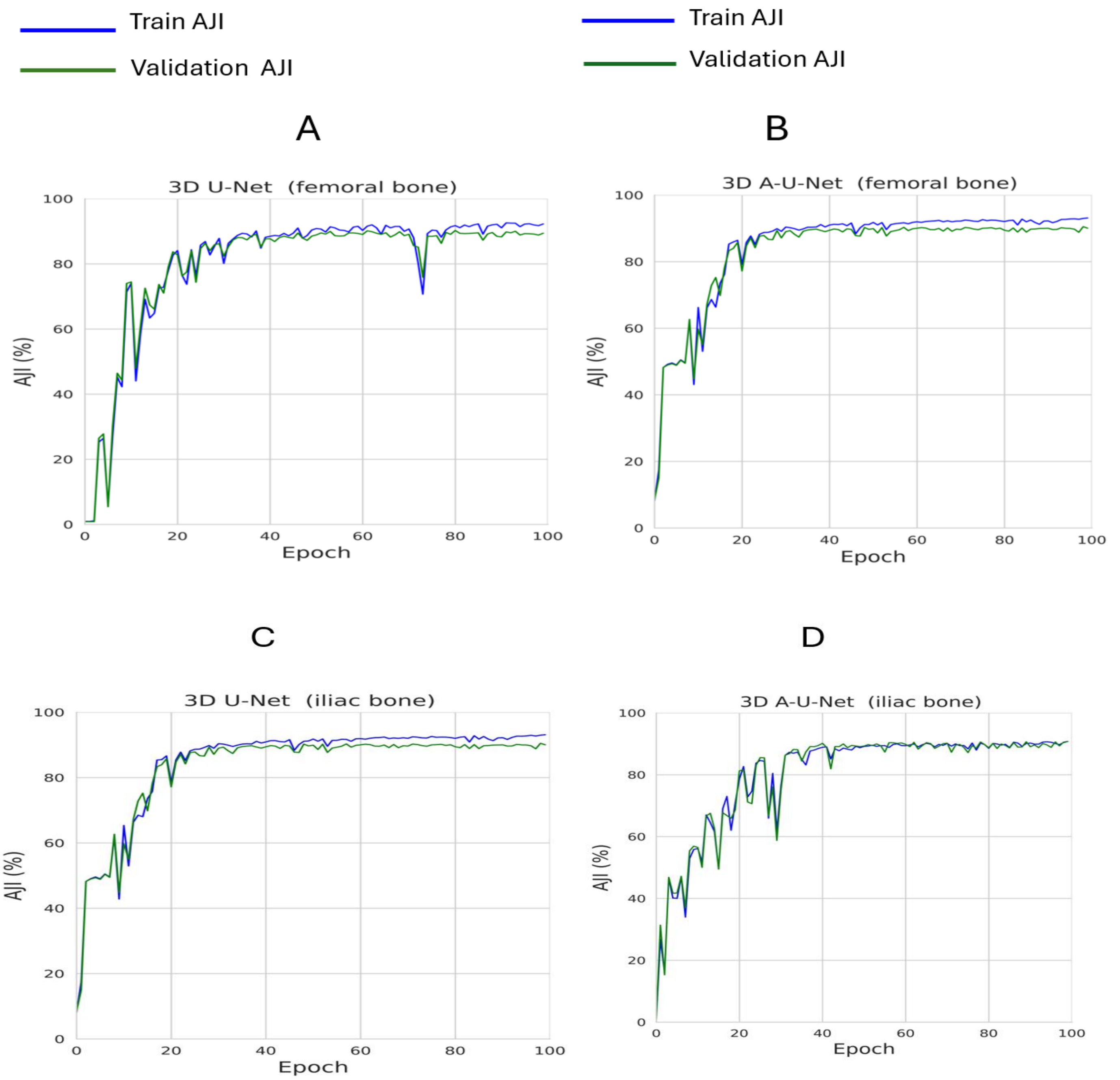

Figure S2. Training and validation performance trends (color-coded in the legend) for 3D U-Net best models (A) femoral bone (3D U-Net) (B) femoral bone (3D A-U-Net) (C) iliac bone (3D U-Net) (D) iliac bone (3D A-U-NET)

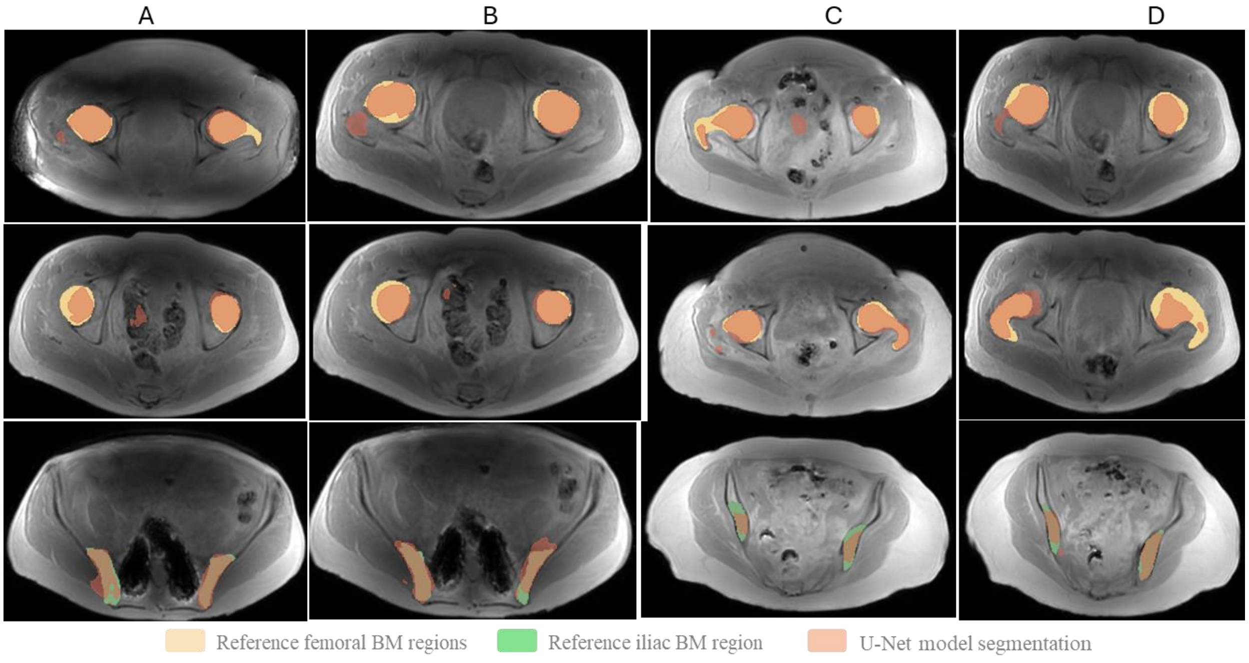

Figure S3. Examples of false positive (FP) and false negative (FN) segmentation errors in femoral BM (top two rows) and iliac BM (bottom row) across four U-Net models **(A)** 2D U-Net **(B)** 2D A-U-Net **(C)** 3D U-Net **(D)** 3D A-U-Net. The reference and model segmentation regions are color-coded in the legend. All U-Net segmentations are overlayed on top of expert regions.

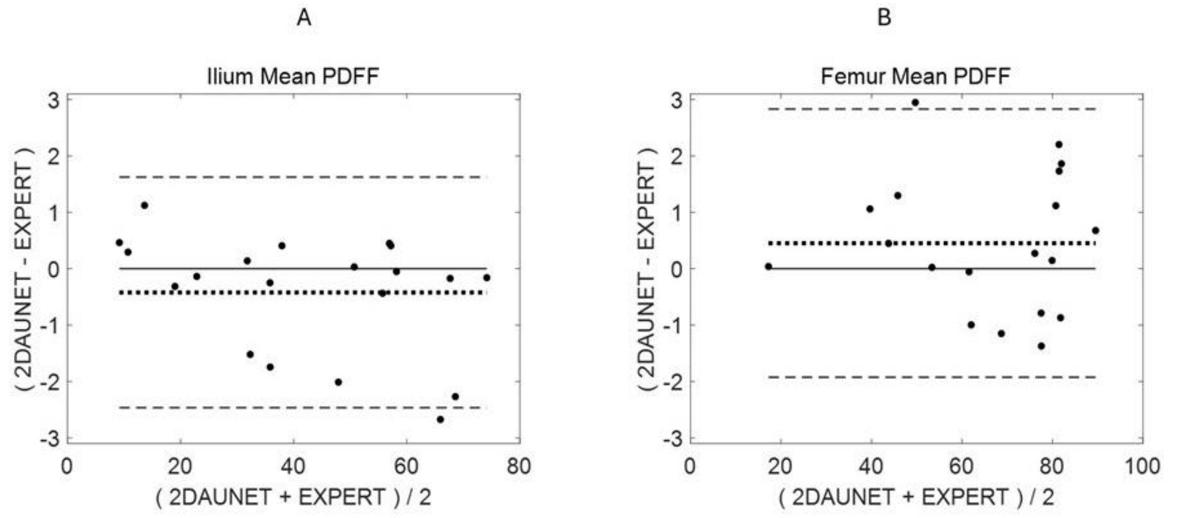

Figure S4: Bland-Altman comparison of quantitative mean proton density fat-fraction (PDFF) via Expert vs 2D A-UNET segmentation of bone marrow within **(A)** Ilium and **(B)** Femur for test-set subjects. As is routine, 3-pixel erosion of Expert and 2D A-UNET

segmentation masks was performed prior to calculation of means to reduce effects of low signal-to-noise cortical bone. Dashed lines represent 95% limits-of-agreement relative to mean bias (dotted line). All axes are in % units.
